# Special Issue: Micro- and Macro-Environmental Factors in Solid Cancers

**DOI:** 10.3390/cells10020247

**Published:** 2021-01-27

**Authors:** Elda Tagliabue

**Affiliations:** Molecular Targeting Unit, Department of Research, Fondazione IRCCS Istituto Nazionale dei Tumori di Milano, 20133 Milan, Italy; elda.tagliabue@istitutotumori.mi.it

Paracrine and endocrine signaling between the tumor and host have been convincingly shown to support tumor progression. As cancer development depends on stochastic mutational events, knowledge regarding the signal(s) that pass between tumor cells, the surrounding microenvironment, and host organs at distant anatomic sites is essential for improving diagnostic, prognostic, and therapeutic approaches.

This Special Issue comprises six papers, including three original articles and three reviews, covering a broad range of topics investigating the host factors that support solid tumors, highlighting the relevance of micro- and macro-environmental factors in solid cancers.

The review by Rybinska et al. [1] skillfully summarizes the current knowledge regarding tumor–adipocyte crosstalk. Recently, adipose tissue has developed from being considered an inert energy storage site to being recognized as an endocrine organ that functions in hematopoiesis, lymphopoiesis, immune function, and reproduction. As associations between obesity and some cancers have been suggested and because some tumors appear to spread to adipose-rich sites, the possibility of adipocytes sustaining the progression of tumors that develop in proximity to these cells, as in breast cancer (BC), has emerged. The analysis of adipocytes surrounding tumors has revealed a lack of lipids and the acquisition of fibroblast-like features, supporting the occurrence of intimate crosstalk between cancer cells and adipocytes. Tumor-modified adipocytes (also known as cancer-associated adipocytes (CAAs)) comprise a large population of stromal cells in BC, which are entirely committed to sustaining tumor progression and promoting resistance to treatment. 

Although the precise mechanism(s) involved in the crosstalk between cancer cells and adipocytes remains to be determined, adipocytes that have previously been conditioned by tumor cells are recognized as releasing molecules that can stimulate tumor invasiveness. Recent evidence has demonstrated that CAAs feature an altered secretome compared with that of mature adipocytes, similar to obese adipocytes, which suggests that the increased tumor incidence in obese people is an effect of altered adipocytes. Thus, the targeting of adipocytes or their crosstalk with tumor cells deserves further investigation to improve tumor therapy and prevention strategies.

The micro-environmental factors that support solid tumors represent a complex network that includes both host cellular and molecular components. The extracellular matrix (ECM) surrounding solid tumors has been reported to play relevant roles in tumor development and progression. The ECM consists of a network of biochemically distinct components, including fibrous proteins, glycoproteins, proteoglycans, and polysaccharides, and the tumor ECM has been shown to differ significantly from that in normal organs. The ECM influences intratumoral signaling, transport mechanisms, and metabolism, as well as affects neoplastic cell dissemination and the development of resistance to therapy. 

Palumbo et al. [2] provide a thorough review of the spectrum of interactions potentially mediated by the ECM in esophageal cancer (EC). EC is a highly lethal cancer, and the current knowledge of EC biology remains limited. Similar to most solid tumors, in EC, the interaction between tumor cells and the ECM governs various phenomena that are crucial for tumor progression. The ECM molecules and signaling pathways that have been identified as being altered in EC include lysyl oxidase (LOX) and the matrix metalloproteinases (MMPs). These proteins are fundamental for collagen turnover and therefore regulate ECM stiffness. Increased ECM stiffness promotes tumor cell growth by inducing telomerase activity, followed by telomere elongation, which sustains a limitless replication pattern that is accompanied by genetic instability. The increased production of ECM molecules has been reported to improve tumor cell adhesion, resulting in the initiation of intracellular signaling pathways, which are crucial for tumor cell survival, migration, and the activation of oncogenic signaling. Conversely, increased MMP levels can be triggered by active signaling pathways in tumor cells, such as the mitogen-activated protein kinase (MAPK) pathway.

This review also focuses on the crosstalk between EC cells and the ECM as a likely mechanism through which risk factors that are associated with EC (obesity and gastroesophageal reflux disease) promote tumor survival. Increases in leptin and interleukin-17 (IL-17) levels, which are induced by these risk factors, support higher MMP expression and activity by promoting the development of an inflammatory environment. As the cellular mechanism that underlies the development of EC has not yet been fully elucidated, the documented function of the ECM during EC progression may represent a basis for improving EC management strategies in the future.

Pancreatic ductal adenocarcinoma (PDAC) is among the tumors with a strong desmoplastic reaction, and is associated with high ECM deposition, which has been found to promote both PDAC aggressiveness and resistance to therapy.

Resovi et al. [3] investigated the therapeutic value of targeting connective tissue growth factor (CCN2/CTGF), a profibrotic matricellular protein that is highly expressed in the PDAC microenvironment and is associated with disease progression. CCN proteins are produced and secreted by many tumors and bind to various ECM molecules and cellular receptors (e.g., Notch, integrins, TrkA, and heparan sulfate proteoglycans) to modulate various activities, including replication, death, adhesion, motility, and ECM production. Given its role in PDAC progression, CCN2 has recently been proposed to serve as a therapeutic target for this aggressive tumor. The authors investigated the therapeutic value of two modified synthetic peptides that were derived from the active regions of CCN3, which is an endogenous inhibitor of CCN2. Peptide treatment in a murine orthotopic PDAC model impaired collagen deposition in the tumor microenvironment and increased chemotherapeutic activity, revealing new therapeutic perspectives for one of the most aggressive tumor types with limited treatment options. 

The tumor stroma and leukocyte infiltration are widely recognized as supporting tumor progression by fueling inflammation, and the administration of non-steroidal anti-inflammatory drugs (NSAIDs) after diagnosis could increase long-term survival. Prostaglandin-endoperoxide synthase-2 (PTGS2), one of the key enzymes that mediate prostaglandin neosynthesis, is typically induced by inflammatory stimuli. PTGS2 is considered an ideal target for reducing tumor inflammation, especially in colorectal cancer (CRC) patients. Despite an ongoing, multicenter, Phase III trial to evaluate the efficacy of a PTGS2 inhibitor, celecoxib, combined with folinic acid, fluorouracil, and oxaliplatin (FOLFOX) chemotherapy, established criteria for patient selection are still lacking. 

By analyzing PTGS2 levels in a series of 100 primary CRC patients using immunohistochemistry (IHC) and immunofluorescence double-staining assays, Venè et al. [4] show that cancer-associated fibroblasts (CAFs) represent a prevalent, non-tumor source of PTGS2. The in vitro analysis of primary CRC-associated CAFs revealed the powerful induction of PTGS2 expression by IL1β treatment, which is an important mediator of the inflammatory response. Intermediate levels of stromal PTGS2 were associated with better prognosis, whereas high PTGS2 levels were associated with a negative outcome compared with low- or null-PTGS2-expressing CRCs. In contrast, tumor-associated PTGS2, assessed by IHC, did not influence patient outcomes. By verifying the importance of the stroma during tumor progression, they identified an unexpected association between stromal PTGS2 levels and patient prognosis, which supports the potential role of stromal PTGS2 as a predictive marker for the response to NSAID treatment.

Cosentino et al. [5] provide evidence that BC can corrupt the surrounding microenvironment through the release of microRNAs (miRNAs) into the stroma. Specifically, miR-9, released by triple-negative BC (TNBC) cells, was found to perturb the transcriptional landscape of fibroblasts, inducing a shift toward a CAF malignant phenotype through the downregulation of the epidermal growth factor (EGF)-containing fibulin extracellular matrix protein 1 (EFEMP1). TNBC cells that were conditioned with the supernatant of normal fibroblasts that had been transfected with miR-9 or silenced for EFEMP1 became more resistant to cisplatin treatment. EFEMP1 is a structural protein that also interacts with the tissue inhibitor of metalloproteinase-3 (TIMP-3), which, in turn, inhibits the metalloproteinases MMP2 and MMP9, which are highly expressed in breast cancers and are actively involved in matrix remodeling. This study highlights the relevance of miRNAs in tumor cell communication with the host. In addition, because released miRNAs can enter the blood, they provide compelling support that tumors can corrupt the host. 

The cancer cells within a tumor are now recognized as consisting of a heterogenous population, and a small subset of cancer cells, cancer stem cells (CSCs), has been identified as a reservoir of self-sustaining cells for tumor maintenance. Therefore, understanding the interactions between CSCs and the micro- and macro-environment is necessary for designing innovative treatments that are aimed at fighting the source of cancer. As immune tolerance is a key step in cancer development and progression, CSCs adopt specific strategies to escape attack by host immune cells, as reviewed by Castagnoli et al. [6]. Two primary mechanisms that are used by CSCs to prevent attack by immune cells include the downregulation of proteins that are involved in antigen presentation and the upregulation of immune checkpoint molecules, which impair the activity of immune cells. 

CSCs can also contribute to the process of immunoediting by releasing cytokines that modulate the activity of tumor-infiltrating immune cells. In addition, CSCs were found to produce small molecules with immune-suppressive action, including prostaglandin E2, which induces a shift from a Th-1 to a Th-2 immune response. The immune checkpoint molecules that are expressed by CSCs not only inhibit immune system activation but also sustain tumor stemness by stimulating the expression of molecules that are associated with CSC-specific signaling. Soluble inflammatory cytokines that are released into the tumor microenvironment by tumor-infiltrating immune cells can also modulate CSC activities. Thus, the dynamic crosstalk that occurs between CSCs and the immune tumor microenvironment represents a key player in allowing CSCs to escape host immune recognition, sustaining tumor maintenance and expansion. These results support several ongoing trials that are testing the efficacy of combining therapies against CSCs with existing therapies that use immune-modulating agents that promote an effective antitumor immune response. 

Recognizing that many aspects of tumor biology can be explained by the dynamic crosstalk that occurs between the tumor and the host, we hope that this Special Issue will be of interest, particularly to researchers who are focused on tumor–host interactions. The findings from these studies are expected to provide a better understanding of how local and systemic environments contribute to cancer progression and to serve as the basis for novel diagnostic and therapeutic approaches.

## References

[1] Rybinska I., Agresti R., Trapani A., Tagliabue E., Triulzi T. (2020). Adipocytes in Breast Cancer, the Thick and the Thin. Cells.

[2] Palumbo A., Meireles Da Costa N., Pontes B., Leite de Oliveira F., Lohan Codeco M., Ribeiro Pinto L.F., Nasciutti L.E. (2020). Esophageal Cancer Development: Crucial Clues Arising from the Extracellular Matrix. Cells.

[3] Resovi A., Borsotti P., Ceruti T., Passoni A., Zucchetti M., Berndt A., Riser B.L., Taraboletti G., Belotti D. (2020). CCN-Based Therapeutic Peptides Modify Pancreatic Ductal Adenocarcinoma Microenvironment and Decrease Tumor Growth in Combination with Chemotherapy. Cells.

[4] Vene R., Costa D., Augugliaro R., Carlone S., Scabini S., Casoni P.G., Boggio M., Zupo S., Grillo F., Mastracci L. (2020). Evaluation of Glycosylated PTGS2 in Colorectal Cancer for NSAIDS-Based Adjuvant Therapy. Cells.

[5] Cosentino G., Romero-Cordoba S., Plantamura I., Cataldo A., Iorio M.V. (2020). miR-9-Mediated Inhibition of EFEMP1 Contributes to the Acquisition of Pro-Tumoral Properties in Normal Fibroblasts. Cells.

[6] Castagnoli L., De Santis F., Volpari T., Vernieri C., Tagliabue E., Di Nicola M., Pupa S.M. (2020). Cancer Stem Cells: Devil or Savior-Looking behind the Scenes of Immunotherapy Failure. Cells.

